# Assessment of AAV Dual Vector Safety in the *Abca4^−^^/^^−^* Mouse Model of Stargardt Disease

**DOI:** 10.1167/tvst.9.7.20

**Published:** 2020-06-18

**Authors:** Michelle E. McClements, Alun R. Barnard, Peter Charbel Issa, Robert E. MacLaren

**Affiliations:** 1 University of Oxford, Nuffield Department of Ophthalmology, Clinical Neurosciences, Oxford, UK; 2 Oxford Eye Hospital, Oxford, UK

**Keywords:** dual vector, AAV, ABCA4, Stargardt disease, safety

## Abstract

**Purpose:**

Adeno-associated viral (AAV) gene therapy treatment for Stargardt disease currently requires a dual vector approach owing to the size of the ATP-binding cassette transporter family member gene (*ABCA4*). The nature of the dual vector system creates the potential for adverse events. Here we have investigated an overlapping adeno-associated viral *ABCA4* dual vector system for signs of toxicity in *Abca4^−^^/^^−^* mice as a prelude to dual vector first in human clinical trials.

**Methods:**

*Abca4^−^^/^^−^* mice received a subretinal injection of a 1:1 5′:3′ dual vector mix; 5′ vector only; 3′ vector only; a GFP reporter vector; or diluent only (sham). All vectors were adeno-associated virus-8 Y733F. Mice were subsequently assessed for signs of toxicity as measured by loss in retinal structure by optical coherence tomography and retinal function by electroretinography up to 6 months after injection.

**Results:**

Subretinal delivery of the dual vector system and its comprising parts induced no structural or functional changes relative to paired uninjected eyes beyond those observed in the sham control cohort. Histologic changes were limited to the superior retina where the injection was performed. Electroretinography analysis confirmed the dual vector system inferred no functional changes beyond those observed in the sham control cohort.

**Conclusions:**

An optimized overlapping dual vector system for the treatment of Stargardt disease shows no additional signs of toxicity beyond those observed from a sham injection.

**Translational Relevance:**

This presentation of safety of a dual vector system for the treatment of Stargardt disease encourages its future use in clinical trial.

## Introduction

**Figure 1.**
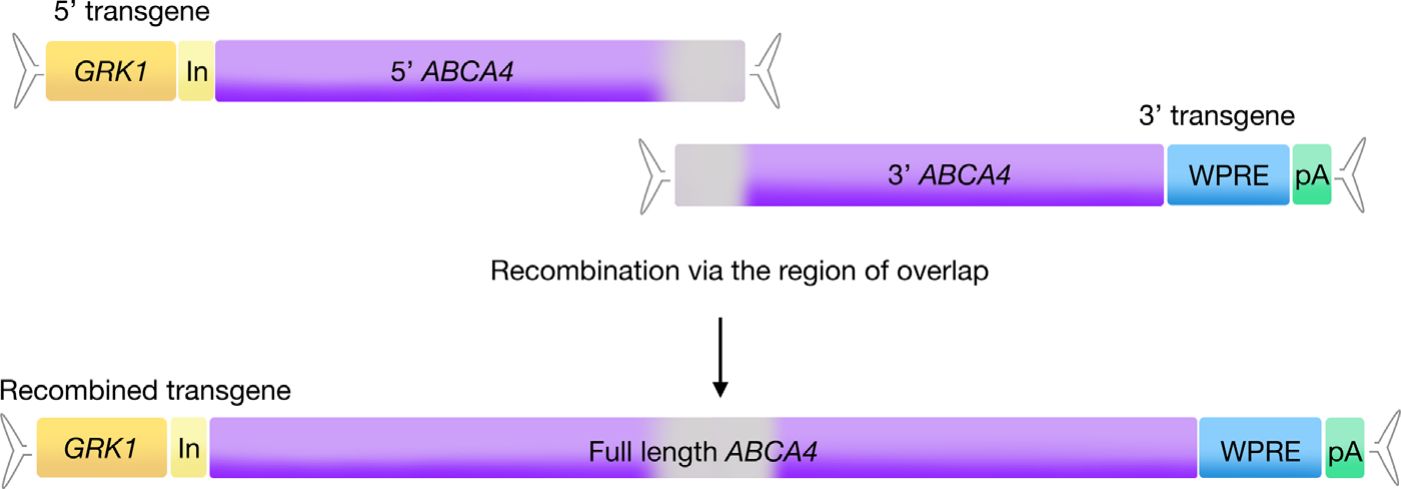
Overview of the *ABCA4* dual transgene system. The genetic elements of a typical AAV transgene are split across two transgenes, named 5′ and 3′. The 5′ transgene contains the promoter and 5′ fragment of *ABCA4* coding sequence, whereas the 3′ transgene carries a 3′ fragment of *ABCA4* coding sequence plus a WPRE and bovine growth hormone polyA signal. Once inside the same host cell nucleus, the two transgenes align and recombine via a region of homology shared between the transgenes (*grey*). ABCA4, ATP-binding cassette transporter protein family member 4; GRK1 = human rhodopsin kinase promoter; In, intron; pA, polyA signal; WPRE, Woodchuck hepatitis virus post-transcriptional regulatory element.

With growing evidence of adeno-associated viral (AAV) vector safety and efficacy in clinical trials for inherited retinal disease,^1^^–^^4^ developing AAV strategies for large gene disorders that currently have no approved pharmaceutical treatment options seems to be worthwhile. Dual vector AAV strategies have shown promise for many years^5^^–^^9^ and the approaches and mechanisms of dual vector success have been discussed elsewhere.^10^^,^^11^ Briefly, the overlapping dual vector approach requires that the basic elements of an AAV gene therapy transgene be split across two separate transgenes. The “5′” transgene contains the promoter, short intron, and 5′ portion of gene coding sequence between two inverted terminal repeat sequences, whereas the “3′” transgene contains the 3′ portion of the gene coding sequence, Woodchuck post-transcriptional regulatory element and poly A signal between two inverted terminal repeat sequences (Fig. 1).

Previous dual vector strategies have shown successful production of the desired protein, but have also shown problems in expression levels and generation of truncated protein forms.^4^^,^^5^ After extensive optimization, we have previously shown that an overlapping dual vector system for the treatment of Stargardt disease enabled clearly detectable levels of ABCA4 expression in the photoreceptor outer segments of *Abca4^−^^/^^−^* mice.^9^ The levels of ABCA4 expression achieved following dual vector delivery significantly altered the biochemistry of the injected retina such that reduction in the levels of bisretinoids, which characteristically build up in Stargardt disease, were detected in addition to an associated reduction in 790 nm autofluorescence in treated eyes. Combined, the data indicated that a therapeutic effect was achieved for the treatment of a large gene disorder from an AAV dual vector system. Similar results were recently achieved with a variant dual vector system and confirm the potential of a dual vector system to offer therapeutic effect.^12^ With these studies showing such encouraging data, the dual vector system has demonstrated its potential to make the move to clinical trial but, before doing so, it is important to consider the risks of the strategy. Here we progress investigations using the optimized overlapping AAV dual vector system by assessing whether it induces any adverse structural or functional effects.

Despite an absence of Abca4, *Abca4^−^^/^^−^* mice show no structural or functional signs of retinal degeneration other than age-related changes^13^ and therefore enable assessment of such changes following subretinal injection. Our study included a cohort injected with a GFP reporter vector as a control group to confirm that the measures employed in the study would be able to identify signs of toxicity as GFP delivery by AAV has previously been shown to cause adverse effects to the retina.^14^^,^^15^

All experiments described were conducted concurrently with our previously published work, which described and confirmed the successful optimization and generation of a dual vector system.^9^ Vector preparations described in our previous publication were used for the cohorts included in this report, injections for which were conducted in parallel with our previous study.

## Methods

### Vector Production

Full details of the transgene design are described in a previous publication^9^ but, briefly, the 5′ transgene was created by combining 199 nucleotides of the human rhodopsin kinase promoter (*GRK1*) with 176 nucleotides of the CAG intron region and nucleotides 1 to 3701 of the *ABCA4* coding sequence (NM_000350). The 3′ transgene was created by combining nucleotides 3494 to 6822 of *ABCA4* coding sequence with 593 nucleotides of the Woodchuck hepatitis virus post-transcriptional regulatory element and 269 nucleotides of the bovine growth hormone polyA signal. Each transgene contained AAV2 inverted terminal repeats and was packaged into AAV8 Y733F using a standard PEI triple transfection method of HEK293T cells with 500 µg total DNA made up of pTransgene, pRepCap, and pHelper. Cells were harvested 3 days after transfection, lysed, and the AAV isolated by ultracentrifugation with an iodixanol gradient followed by purification in Amicon Ultra-15 100K filter units (Merck Millipore, Watford, UK). The final preparations were collected in phosphate-buffered saline (PBS). SDS-PAGE analysis confirmed the purity of each preparation and SYBR Green qPCR titers were determined using primers targeting either the 5′ or 3′ portion of *ABCA4* coding sequence.^9^ Before injection, vector preparations were diluted in PBS with 0.001% Pluronic F-68 (Life Technologies Ltd, Paisley, UK), which also served as the injection material for sham injected eyes. Vector preparations were as used in a previous publication.^9^

### Subretinal Injections

All animal breeding and experimental procedures were performed under the approval of local and national ethical and legal authorities and were conducted in compliance with the Association for Research in Vision and Ophthalmology statement for the use of animals in ophthalmic and vision research. Pigmented *Abca4^−^^/^^−^* mice (129S4/SvJae*Abca4*^tm1Ght^) were kindly provided by Gabriel Travis (David Geffen School of Medicine, University of California, Los Angeles, CA) and bred in the Biomedical Sciences Division, University of Oxford. Animals were kept in 12-hour light/dark cycles with food and water available ad libitum. Animals at 4 to 5 weeks of age were anesthetized by intraperitoneal injection containing ketamine (80 mg/kg) and xylazine (1 0mg/kg) and pupils were fully dilated with tropicamide and phenylephrine eye drops (Bausch & Lomb, London, UK). Proxymetacaine eye drops (Bausch & Lomb) were applied before subretinal injection, which were performed under direct visual guidance using an operating microscope (Leica Microsystems, Wetzlar, Germany). An anterior paracentesis was performed with a 33G needle before subretinal injection using a WPI syringe and beveled 35G needle system (World Precision Instruments, Hitchin, UK). After the injection, chloramphenicol eye drops were applied (Bausch & Lomb) and anesthesia was reversed with atipamezole (2 mg/kg) and carbomer gel applied to the eyes to prevent cataract development (Novartis, London, UK).

For the main study, *Abca4^−^^/^^−^* mice were injected at 4 to 5 weeks of age in their right eye only with 2 µL of one of the following: AAV diluent; GFP reporter vector (2E+10gc); 5′ vector low dose (2E+09 gc); 5′ vector high dose (2E+10 gc); 3′ vector (1E+10 gc); dual vector low dose (2E+09 gc); or dual vector high dose (2E+10 gc). All vectors were AAV8 Y733F and full details are provided in Table 1. For the follow-up study, *Abca4^−^^/^^−^* mice were injected at 4 to 5 weeks of age receiving 1 µL of AAV diluent in one eye (sham) and dual vector high dose (2E+10 gc) in the contralateral eye.

**Table 1.** Details for the Materials Injected into *Abca4^−^^/^^−^* Mice

Injection Material Details	Dose (Total Genome Copies Per Eye)	Abbreviated Name
AAV diluent: PBS with 0.001% PF68	Not applicable	Sham
GRK1.GFP.pA: *GRK1* promoter driving GFP expression	2E+10	GFP
GRK1.ABC: *GRK1* promoter with the 5′ portion of *ABCA4* coding sequence	2E+10	5′ vector high dose
	2E+09	5′ vector low dose
CA4.WPRE.pA: 3′ portion of *ABCA4* coding sequence with WPRE and polyA signal	1E+10	3′ vector
GRK1.ABC + CA4.WPRE.pA: 1:1 mix of 5′ and 3′ vectors	2E+10	Dual vector high dose
	2E+09	Dual vector low dose

PF68, pluronic PF-68.

### Optical Coherence Tomography

Optical coherence tomography (OCT) captures cross-sectional images of the retina using low-coherence interferometry of infrared light and images were taken using a 55° lens on a Spectralis HRA-OCT (Heidelberg Engineering, Heidelberg, Germany). Animals were anesthetized and pupils fully dilated as described elsewhere in this article. A custom-made contact lens (Cantor & Nissel Ltd, Brackley, UK) was placed on the cornea with Hypromellose 1% eye drops (Alcon, Camberley, UK) as a viscous coupling fluid. The NIR reflectance image (820-nm diode laser) was used to align the fundus camera relative to the pupil and 488 nm and 790 nm autofluorescence images were taken. A high-resolution radial scan was applied with the OCT function with total retina and outer retina thickness measurements made manually after imaging. A central marker was set and calipers applied at a standardized distance from this point on the Heidelberg software (Supplementary Figure S1). Total retinal thickness was measured from the inner limiting membrane to the inner margin of the retinal pigment epithelium. Outer segment thickness was measured from the external limiting membrane to the inner margin of the retinal pigment epithelium.

### Electroretinography

Animals were dark-adapted for 12 to 16 hours before anesthetizing and dilating pupils fully as described elsewhere in this article. All work was conducted in the dark under dim red light. Each animal was positioned on a heated platform within the testing console (Colordome Electroretinography Machine, Diagnosys LLC, Cambridge, UK). Flash stimuli were delivered in a Ganzfield dome with recordings made in a light-tight Faraday cage. Subcutaneous ground and reference platinum electrodes were placed in the flank and scalp. Custom lenses were produced by attaching a DTL plus silver-coated nylon thread active electron (Diagnosys LLC) to achromatic Aclar embedding (Honeywell International, Charlotte, NC). A lens was positioned concentrically on the cornea of each eye using micromanipulators and viscous coupling media (Hypromellose 1%, Alcon). For scotopic testing, animals were acclimatized to the environment for 10 minutes before initiating the testing protocol (Supplementary Table S1). Animals were then exposed to a full-field 30 cd/m^2^ white background for 10 minutes, a stimulus that was maintained for the subsequent photopic testing steps (Supplementary Table S2). Electroretinogram (ERG) a-wave and b-wave amplitudes were measured manually using Espion v6 software. The a-wave and b-wave cursors were placed manually and full b-wave amplitudes were measured from the trough of the a-wave to the peak of the b-wave.

### Immunohistochemistry

After enucleation, the lens and cornea were removed and each eye cup incubated in 4% PFA, incubated in a 30% sucrose solution before being frozen in OCT compound (VWR, Lutterworth, UK). Eyes were sectioned and dried before permeabilizing with 0.2% Triton-X 100. Slides were washed in PBS before blocking with 10% bovine serum albumin and 10% donkey serum. Slides were washed before primary antibody incubation (goat polyclonal, ABIN343052, AntibodiesOnline [Aachen Germany], used 1/200 in 1% bovine serum albumin, 1% serum) and secondary antibody incubation (donkey anti-goat 488, ab1050129 [Abcam, Cambridge, UK] used 1/400 in 1% bovine serum albumin and 1% serum). Hoescht stain was performed (Sigma-Aldrich, Gillingham, UK) (1/2,000 in PBS) before a final wash in PBS. ProLong Diamond antifade mounting medium (Life Technologies Ltd, Paisley, UK) was applied and slides were sealed before imaging.

### Statistical Analysis

Each dataset was assessed for normal distribution (Shapiro-Wilk test) and variance (Brown-Forsythe test). Data were normally distributed therefore parametric tests were used (analysis of variance). Multiple comparisons were conducted with correction using either the Tukey or Sidak comparisons tests. Figure legends indicate the specific tests used to analyze each dataset. Data are presented as mean and standard error of the mean unless otherwise stated.

## Results

### Comparisons of Structural Damage

All injections were performed in the superior retina and mice in all cohorts suffered varying degrees of damage after subretinal injection that, when compared using a subjective scoring system (examples shown in Supplementary Figure S1), revealed that no one group suffered more damage than any other (Table 2).

**Table 2.** Degree of Observed Damage in All Cohorts

Degree of Damage	Sham	GFP 2E+10	5′ 2E+09	5′ 2E+10	3′ 1E+10	Dual 2E+09	Dual 2E+10	Average
+	5	4	6	3	4	5	6	4.71
++	2	4	3	3	2	1	3	2.57
+++	2	2	1	3	2	3	2	2.14
++++	2	0	0	1	0	1	0	0.57
Total	11	10	10	10	8	10	11	–
Χ^2^	0.2927	0.6862	0.6587	0.7151	0.8455	0.6499	0.8005	–

+, least damage; ++++, most damage.

Example images relating to the scoring system applied are shown in Supplementary Figure S1.

**Figure 2.**
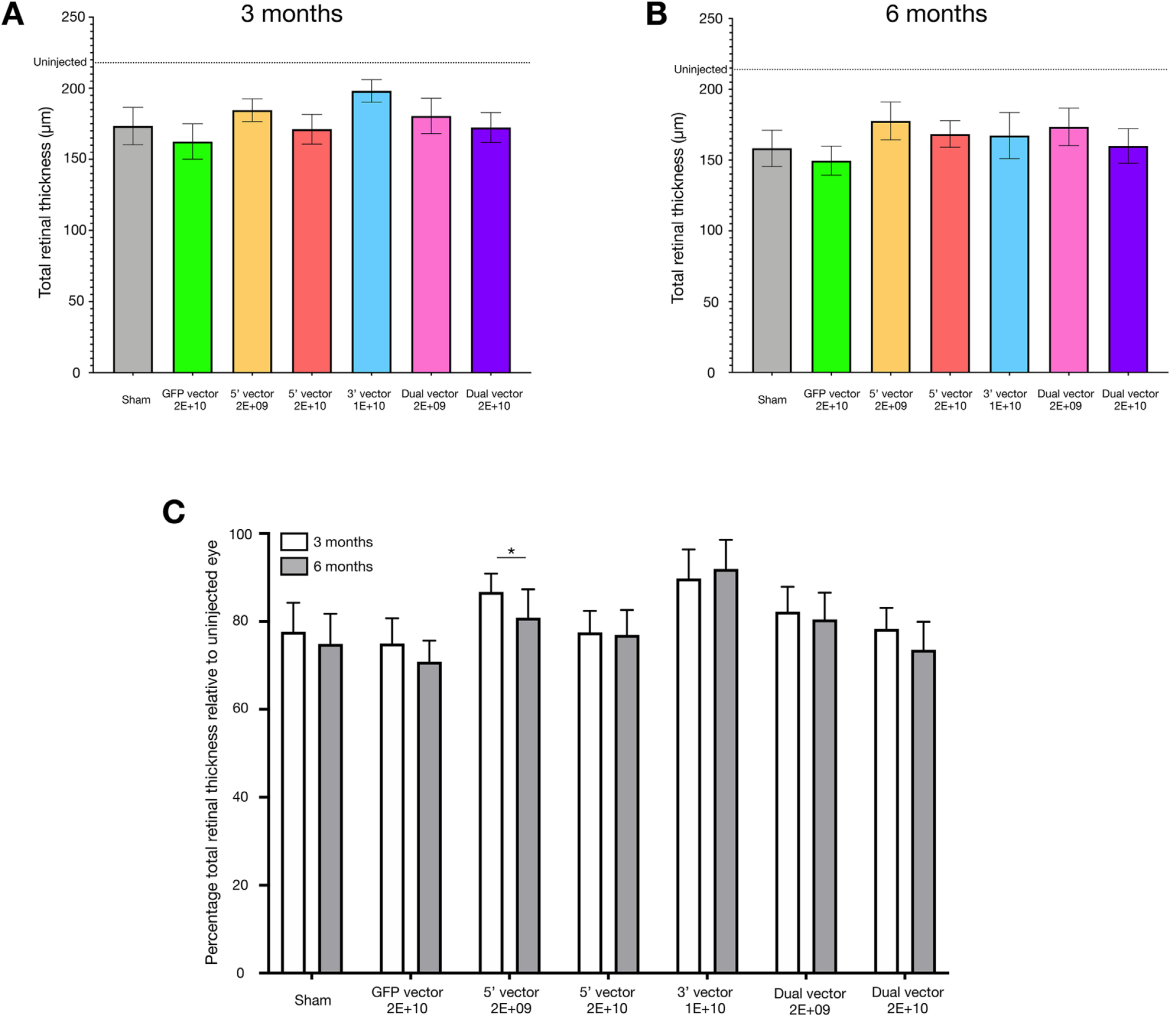
Total retinal thickness measurements at the area of injection for all cohorts at 3 months (A) and 6 months (B) after injection. The average total retinal thickness of uninjected eyes is marked for reference. All cohorts showed similar changes in total retinal thickness at 3 months (one-way analysis of variance; *P* = 0.5358, F = 0.8539) and 6 months (one-way analysis of variance; *P* = 0.7104, F = 0.6238). Normalizing the measurements to those of the uninjected eye (C) revealed the changes in total retinal thickness for each cohort were not significantly influenced by the injection material, but were influenced by the time point of measurement (two-way analysis of variance, injection material *P* = 0.6227, time point *P* = 0.0030, interaction *P* = 0.2415), although only the 5′ 2E+09 cohort underwent a significant change in total retinal thickness between 3 and 6 months (Sidak's multiple comparisons; *P* = 0.0320). For dataset A: sham = 8, GFP = 7, 5′ vector 2E+09 = 9, 5′ vector 2E+10 = 7, 3′ vector = 5, dual vector 2E+09 = 9, and dual vector 2E+10 = 7. For dataset B: sham = 10, GFP = 10, 5′ vector 2E+09 = 8, 5′ vector 2E+10 = 9, 3′ vector = 6, dual vector 2E+09 = 9, and dual vector 2E+10 = 10. For dataset C, only mice for which the 3- and 6-month data were available were included: sham = 7, GFP = 7, 5′ vector 2E+09 = 7, 5′ vector 2E+10 = 7, 3′ vector = 3, dual vector 2E+09 = 9, and dual vector 2E+10 = 7.

For quantitative comparisons of retinal damage between cohorts, OCT measurements were taken at three defined locations in both the superior and inferior retina (Supplementary Figure S2). Retinal thickness measurements were assessed at 3 and 6 months after injection with minimal variation in the measurements of the inferior retina at both time points (Supplementary Figure S3). All injected eyes showed a decreased total retinal thickness in the area of injection, but these changes were not significantly different between the cohorts (Fig. 2). All cohorts suffered a 10% to 25% loss in total retinal thickness of the injected area of retina compared with paired uninjected eyes, which was largely attributed to a loss in outer segment thickness (Supplementary Figure S4, Supplementary Table S3). Example OCT sections for injected eyes in each cohort are shown in Supplementary Figure S5.

The extent of the structural changes between paired injected and uninjected eyes showed no significant differences between cohorts within each time point. The degree of loss in total retinal thickness between paired uninjected and injected eyes in the area of injection was observed to significantly change over time (Fig. 2C; *P* = 0.0030) but only the 5′ 2E+09 cohort showed a significant difference in the magnitude of loss in total retinal thickness compared with paired uninjected eyes between 3 and 6 months after injection (*P* = 0.0320). These comparisons indicated that the majority of structural changes occurred in the first 3 months after injection and subsequent changes in structure were in line with the age-related loss that occurred in the paired uninjected eyes. The significant difference in the 5′ 2E+09 cohort was of surprise, because the degree of loss in retinal structure in the injected eyes of this cohort was typically lower than other cohorts. Despite the significant change over time, the degree in the loss of structure compared with paired uninjected eyes was still less than that observed in the sham injected cohort. Additionally, no such difference was observed in the cohort that received 5′ 2E+10. If a toxic effect were occurring owing to this 5′ vector/transgene it would be anticipated that a greater degree of difference would be observed in the higher dose cohort, but this was not the case.

Observations of cell presence in the vitreous were also made from the OCT scans. Of the 70 mice injected, six presented indications of cells in the vitreous that were not observed in paired uninjected eyes (three from the cohort 5′ 2E+10 and one each from the sham, GFP, and 2E+09 dual vector cohorts). Observations of cell presence in the vitreous at 3 months were less apparent at 6 months (Supplementary Figure S6).

### Comparisons of Retinal Function

**Figure 3.**
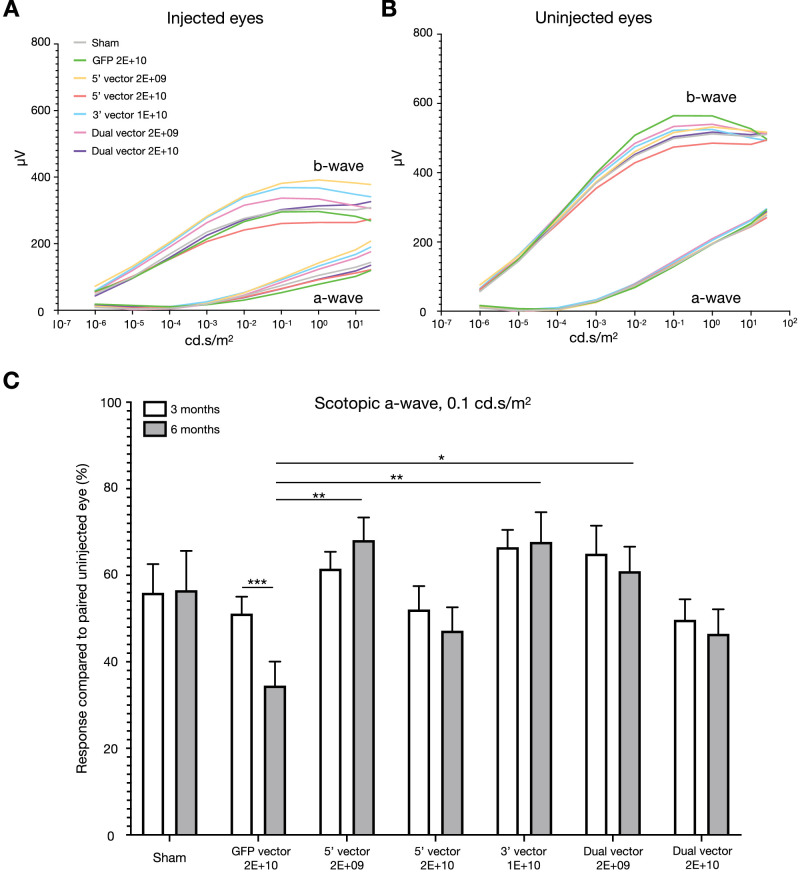
ERG response data 6 months after injection. Smoothed average scotopic a- and b-wave response curves are shown for injected (A) and uninjected (B) eyes for all cohorts. (C) Scotopic a-wave responses at 0.1 cd.s/m^2^ were maintained in injected eyes compared with uninjected eyes between 3 and 6 months after injection except for the GFP cohort (repeated measures two-way analysis of variance, injection material *P* = 0.0200, time point *P* = 0.0695, interaction *P* = 0.0095). For the GFP-injected mice, there was a significant reduction in the response of the injected eye between 3 and 6 months after injection (Sidak's multiple comparisons; *P* = 0.0007). The GFP cohort responses were also significantly different to other cohorts at 6 months after injection (Tukey's multiple comparisons, 5′ vector 2E+09; *P* = 0.0019; 3′ vector 1E+10; *P* = 0.0035, dual vector 2E+09; *P* = 0.0315). For datasets A and B: sham = 11, GFP = 10, 5′ vector 2E+09 = 10, 5′ vector 2E+10 = 10, 3′ vector = 8, dual vector 2E+09 = 9, and dual vector 2E+10 = 10. For dataset C, only mice for which the 3- and 6-month data were available were included: sham = 11, GFP = 10, 5′ vector 2E+09 = 9, 5′ vector 2E+10 = 8, 3′ vector = 8, dual vector 2E+09 = 9, and dual vector 2E+10 = 10.

ERG assessments were conducted for all cohorts at 3 and 6 months after injection. The average scotopic a- and b-wave responses for injected and uninjected eyes in each cohort are displayed in Figure 3 (6 months) and Supplementary Figure S7 (3 months).

Transgene-induced toxicity could be anticipated to cause continued damage over time; therefore, it was important to assess changes in the degree of loss in retinal function over an extended period. The scotopic a-wave at 0.1 cd.s/m^2^ represents the rod photoreceptor-related response to the visual stimulus. Data were extracted with the amplitude of each injected eye normalized to the response in the paired uninjected eye. The percentage loss of function was then compared between the groups.

At 3 months after injection, all cohorts that received a total dose of 2E+10 genome copies (GFP, 5′, and dual vectors) exhibited on average 68% or less scotopic a-wave function in their injected eye relative to the paired uninjected eye whereas for all other cohorts, function of the injected eye was greater than 68% (Fig. 3C). No significant differences were observed between the cohorts at 3 months after injection. However, remaining scotopic a-wave function of injected eyes in the GFP cohort were significantly different to the 5′ vector 2E+09 (*P* = 0.0019), 3′ vector 1E+10 (*P* = 0.0035) and dual vector 2E+09 (*P* = 0.0315) cohorts at 6 months after injection. The degree of function remaining at 6 months after injection was also significantly different from the 3-month time point for the GFP cohort (*P* = 0.0007) only. This observation was critical because it indicated that, after the initial changes (detected at 3 months after injection), further loss in function of injected eyes were equivalent to age-related changes in paired uninjected eyes for all cohorts except the GFP cohort. No other differences in ERG responses (scotopic b-wave or photopic flash) were observed between the cohorts.

After the 6 months postinjection assessments, ABCA4 presence was confirmed in the photoreceptor outer segments of dual vector injected eyes with an absence of staining observed in other cohorts (Supplementary Figure S8).

### Paired Injected Eyes

**Figure 4.**
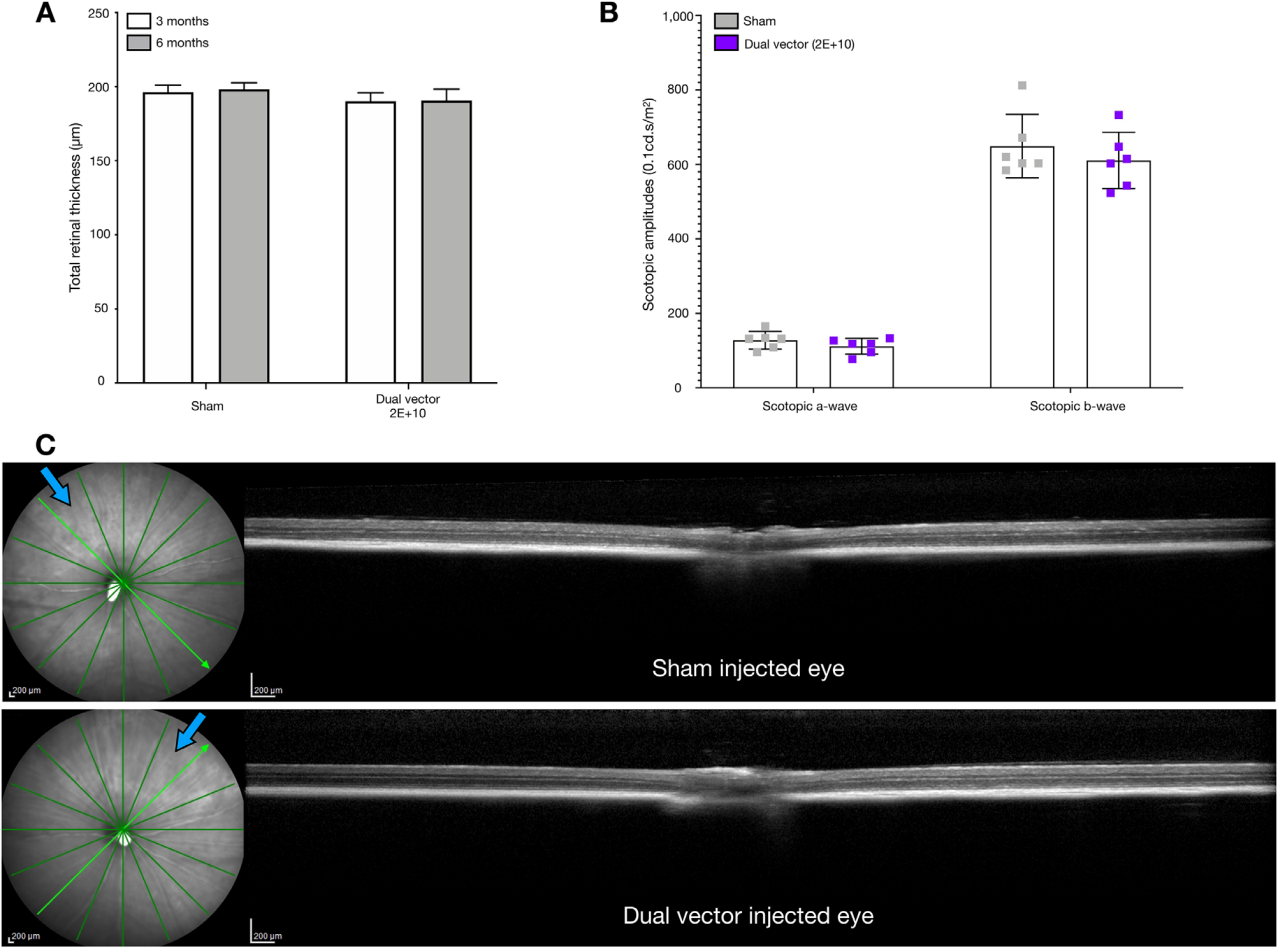
(A) Total retinal thickness measurements of the injection area were equivalent in *Abca4^−^^/^^−^* mice that received a sham injection in one eye and dual vector (2E+10 gc) in the contralateral eye 3 and 6 months after injection (*n* = 6). (B) No significant differences in scotopic a-wave and b-wave (0.1 cd.s/m^2^) amplitudes were identified between paired injected eyes that received either sham injection or dual vector at 5 months after injection (*n* = 6, two-way analysis of variance with Sidak's multiple comparisons: scotopic a-wave *P* = 0.862, scotopic b-wave *P* = 0.4742). (C) Representative OCT sections through the region of subretinal injection in paired sham (*top*) and dual vector (*bottom*) injected eyes. *Blue arrows* indicate the approximate location of the needle when performing subretinal injection.

Combined, the data from this study indicated that, under the conditions used, no significant influence of the injection material or dose of AAV on retinal structure or function could be determined except from the GFP cohort. To confirm a lack of toxicity of the dual vector treatment dose (2E+10 gc) compared with sham injected eyes, a further cohort of *Abca4^−^^/^^−^* mice received a subretinal injection of AAV diluent in one eye and dual vector (2E+10 gc) in the contralateral eye. Paired eyes exhibited limited signs of structural damage with no significant differences in total retinal thickness at 3 or 6 months after injection and no changes in thickness measurements over time (Fig. 4). Furthermore, ERG analysis identified no significant differences in responses, including scotopic a-wave or b-wave 0.1 cd.s/m^2^ amplitudes, between paired eyes at 5 months after injection (Fig. 4B).

## Discussion

In this study, we have demonstrated a lack of toxicity of the *ABCA4* dual vector system. The pigmented *Abca4^−^^/^^−^* mouse model exhibits no histologic or functional signs of retinal degeneration^13^ and, therefore, is a robust model for the assessment of toxicity or any other induced changes after injection. Our study identified no significant differences between the loss in retinal structure and function from the sham-injected cohort to the cohorts that received the dual vector system or its comprising parts at any dose. This finding was confirmed in a follow-up study in which *Abca4^−^^/^^−^* mice received a sham injection in one eye and the high-dose dual vector system in the contralateral eye, with no differences in structure or function measurements observed between paired eyes.

Predicted risks of the dual vector system arise from (A) the potential for unwanted expression products from unrecombined 5′ and 3′ transgenes and (B) the high vector dose used to achieve a therapeutic effect. Regarding risk (A), our previous investigations failed to detect truncated ABCA4 protein following transgene optimizations.^9^ However, *ABCA4* mRNA transcripts from 5′ and 3′ vector only injected eyes were detected; therefore, it is known that some degree of expression occurs from unrecombined transgenes. The 5′ vector contains the *GRK1* promoter, restricting transgene expression to the rod and cone photoreceptors. The 3′ vector contains no promoter therefore expression products are most likely derived from inverted terminal repeat sequence-driven transcription, which would not be cell specific.

Regarding risk (B), single gene therapies typically provide up to 2E+09 genome copies per eye, but we previously observed therapeutic outcomes in the *Abca4^−^^/^^−^* mouse model using 2E+10 total genome copies of a 1:1 5′:3′ dual vector mix. There is a risk that too much AAV could lead to toxicity of the retina.^14^^,^^15^ Both risks (A) and (B) could be anticipated to lead to changes in cell function and, therefore, retinal function and potentially loss of cells, resulting in changes to the retinal structure. Adverse effects of vector dose could be expected to occur within the first few weeks after injection, whereas transgene toxicity could be predicted to continue over time with a greater effect observed at a later time point.

In consideration of the potential for transgene toxicity and/or AAV dose toxicity, we compared structural changes and functional responses in multiple cohorts. To confirm our testing methods were appropriate for identification of toxic responses, we included a cohort that received a high dose (2E+10 genome copies per eye) of AAV8 Y733F containing the reporter transgene GRK1.GFP.pA. Similar transgenes and doses have been shown to induce adverse effects in mice^14^^,^^15^ and, indeed, in our own study, this was the only cohort to show a significant sign of vector-related toxicity. It is of interest that, whereas GFP-injected eyes consistently showed the greatest loss in photoreceptor outer segment thickness and total retinal thickness, these structural differences were not significant when compared with the other cohorts. This finding indicates that assessments of structural changes by OCT thickness would not alone be appropriate to confirm an absence of toxicity following subretinal injection. Given the disruption to the photoreceptor outer segment layer in all injected eyes, the subsequent detection of loss in ERG function was to be anticipated. Our data analysis indicates that the rod photoreceptor response, as measured by the scotopic a-wave, was significantly influenced only in the GFP injected cohort over time. Such a difference was not identified in any other cohort and confirms the ERG measurements used were sensitive enough to detect a difference in photoreceptor response. It is of further interest that the broader bipolar cell response, measured by the scotopic b-wave, did not significantly change over time in any of the cohorts.

A previous report revealed no signs of toxicity when using a photoreceptor-specific promoter with delivery of an AAV8 GFP vector at a dose of 2E+09 gc/eye.^15^ However, there were multiple differences in the conditions used in this study to our own. We delivered a higher dose of a mutant AAV8 serotype (compared with AAV8) into adult mice (compared with P0) and assessed for signs of toxicity at a much later time point of 6 months (compared with 1 month). Indeed at 3 months, our assessments showed no significant signs of toxicity between the cohorts. Combined, these factors will contribute to the differences in results achieved. In line with our data, extensive toxicity was described from a photoreceptor-specific GFP AAV8 vector after delivery of a very high dose (1E+11 genome copies) in adult mice.^14^

The previously optimized *ABCA4* AAV dual vector system^9^ used a therapeutic dose of 2E+10 gc/eye, which was therefore an important dose to be applied in this study. Previous investigations revealed the 5′ vector of the dual vector system does not produce detectable forms of truncated ABCA4 protein and the study described here indicates that if any protein is generated from this vector, it does not cause any adverse effects beyond those observed in a sham injected cohort. Two doses of 5′ vector were investigated and given the expected absence of a transgene protein product from this vector, the 2E+10 gc/eye 5′ vector cohort was intended to act as a control cohort for the influence of the high dose of AAV delivered to the retina. The structural or functional changes observed in this group after injection were not significantly different to those detected in the cohort that received 2E+09 gc/eye of the same vector or indeed the sham injected group. Similarly, for the dual vector injected cohorts, the group that received the higher dose of AAV showed no additional signs of toxicity beyond those observed in other cohorts, including the sham injected group.

It should be noted that, at 3 months after injection, ERG function was decreased in all injected eyes compared with paired uninjected eyes. Although loss in scotopic a-wave function was not significantly different between the cohorts, there was a trend for cohorts that received a 2E+10gc dose of vector to suffer greater loss in function, regardless of the transgene. The same trend was maintained at 6 months after injection, but with significant differences between the GFP 2E+10 cohort and the 5′ vector 2E+09, dual vector 2E+09 and 3′ vector 1E+10 cohorts observed. It is also critical to note that no significant change in function was observed between the time points within each cohort except for the GFP group. This finding is important because it indicates the changes in retinal function that occurred in injected eyes between 3 and 6 months were equivalent to the age-related changes that occurred in paired uninjected eyes over the same time period in all but the GFP cohort.

The identification of a significant change in scotopic a-wave function at 6 months and between time points in the GFP injected cohort only indicates the testing methods and study duration applied were appropriate for detecting adverse effects following subretinal injection. An initial loss of retinal structure was observed in all cohorts, primarily owing to a loss of outer segments after injection, which would explain the loss in ERG output observed compared with uninjected eyes. However, subsequent internal changes to photoreceptor cell health, for example, owing to toxic transgene expression, would not necessarily change the structure of the retina, but would influence the function of the photoreceptor cells. This finding could account for the lack of significant differences between 3 and 6 months in the retinal structure. The combination of these assessments indicate that were the dual vector system and/or its comprising parts to cause such adverse effects, we would likely have detected such changes. Given that three different cohorts received 2E+10 genome copies of an AAV8 Y733F vector per eye yet only the GFP cohort exhibited signs of toxicity, it would seem that the capsid and dose were not the primary causes of the changes observed. Long-term AAV8 Y733F toxicity in the retina has not previously been reported and whilst this study indicates it is safe at a high dose in *Abca4^−^^/^^−^* mice, this may not be true in other strains or models. Any changes in retinal structure and/or function observed at 3 months after injection were maintained at 6 months after injection, without significant further loss except in the GFP injected cohort, which highlights the importance of assessing changes over time in the same mice and including later time points as transgene toxicity may take time to accumulate and become detectable. Although we extended our investigations beyond time frames used in other studies,^14^^,^^15^ it would be of interest to assess changes beyond 6 months after injection.

The 3′ vector of the *ABCA4* AAV dual vector system was previously optimized to decrease the levels of truncated ABCA4 protein generated from the transgene to undetectable levels.^9^ This vector was tested only at the dose provided in the therapeutic dual vector mix (1E+10 gc/eye) and no additional adverse effects were observed beyond those achieved in the sham injected cohort. This was very encouraging as it is expected that only a proportion of the 5′ and 3′ transgenes that comprise the dual vector mix will recombine. It, therefore, has to be anticipated that some 5′ and 3′ transgenes will exist as independent structures in transduced cells. It is worth considering that the doses used in this study for testing single vector toxicity included doses that would provide more single vector transgenes than would likely exist as independent forms in a dual vector injected eye owing to a subpopulation undergoing recombination. Encouragingly, subretinal delivery of the single vectors alone did not result in any significant changes to the structure or function of the retina beyond those observed in sham injected eyes.

To strengthen our claim that the dual vector system confers no further adverse effects beyond that of a sham injection, we tested a final cohort of *Abca4^−^^/^^−^* mice that received sham injection in one eye and dual vector (2E+10 gc/eye) in the contralateral eye. Measurements of retinal structure and function of this cohort confirmed no significant differences between the paired eyes. Combined, our data indicate that the dual vector system generated for the treatment of Stargardt disease induces no adverse effects beyond those observed in sham injected eyes.

## Supplementary Material

Supplement 1

Supplement 2

Supplement 3

Supplement 4

Supplement 5

Supplement 6

Supplement 7

Supplement 8

Supplement 9

Supplement 10

Supplement 11
