# Supplementary material for: Assessment of AAV Dual Vector Safety in the Abca4−/− Mouse Model of Stargardt Disease
Source: Transl Vis Sci Technol. 2020 Jun 18;9(7):20. doi: 10.1167/tvst.9.7.20 (PMC7115835; doi:10.1167/tvst.9.7.20)
Supplement: Supplement 2 [file tvst-9-7-20_s002.pdf]

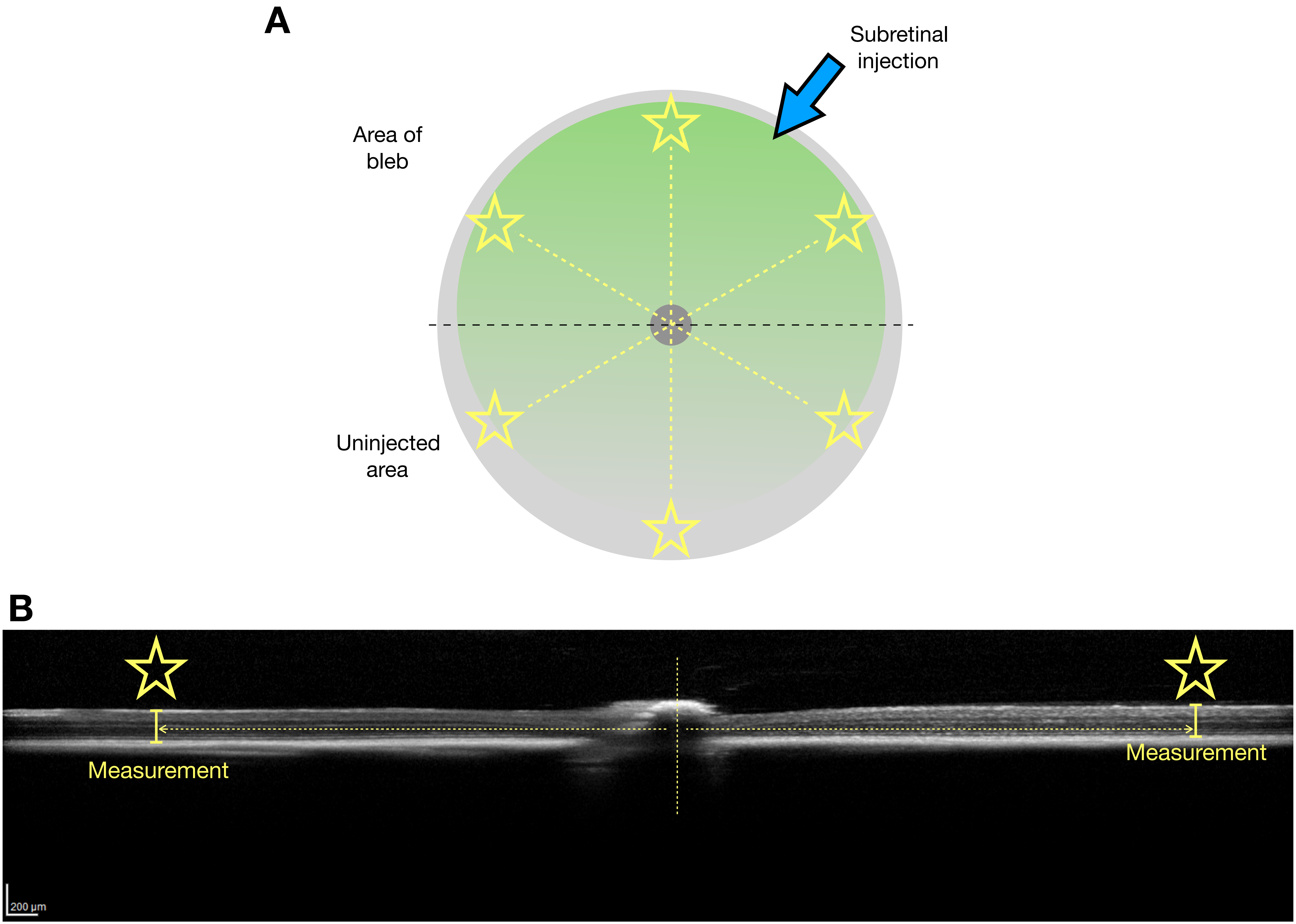

Supplementary Figure 2. For standardized retinal thickness measurements by optical coherence tomography (OCT), radial scans were performed and six defined areas were selected for measurement extraction for each eye. The thickness measurements at three locations were averaged for retinal thickness measurements at the area of injection and uninjected area.
