# Supplementary material for: Assessment of AAV Dual Vector Safety in the Abca4−/− Mouse Model of Stargardt Disease
Source: Transl Vis Sci Technol. 2020 Jun 18;9(7):20. doi: 10.1167/tvst.9.7.20 (PMC7115835; doi:10.1167/tvst.9.7.20)
Supplement: Supplement 3 [file tvst-9-7-20_s003.pdf]

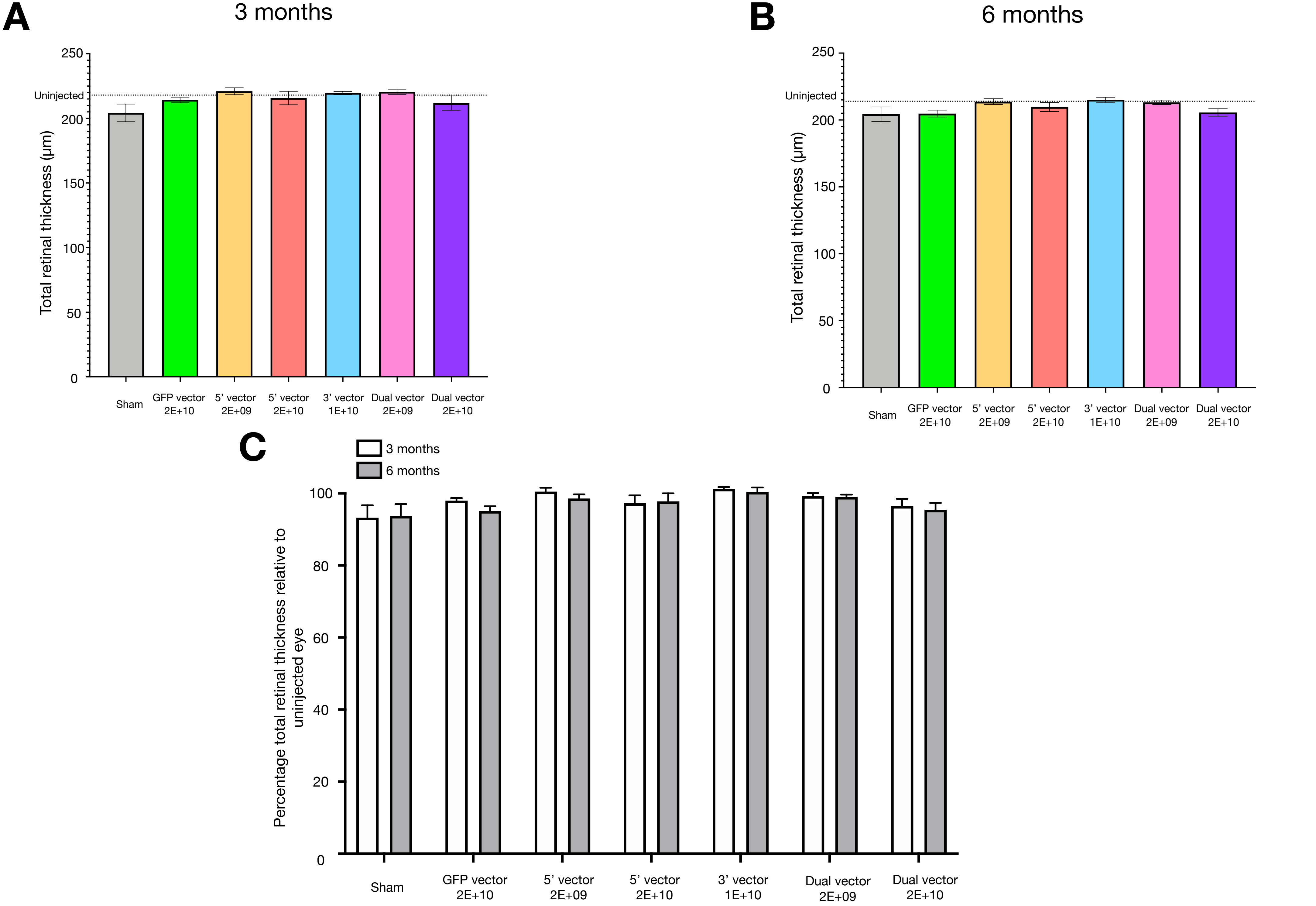

Supplementary Figure 3. Total retinal thickness measurements for the uninjected area of retina of the injected eyes for all cohorts at 3 months (A) and 6 months (B) post-injection. The average total retinal thickness of uninjected eyes is marked for reference. Normalizing the measurements to the equivalent area of the paired uninjected eye (C) revealed no significant changes between cohorts or over time (two-way repeated measures ANOVA, injection material $p=0.1874$, time point $p=0.0516$, interaction $p=0.2071$, no significant differences with Sidak’s multiple comparisons). For data set A : sham = 8, GFP = 7, 5’ vector 2E+09 = 9, 5’ vector 2E+10 = 7, 3’ vector = 5, dual vector 2E+09 = 9, Dual vector 2E+10 = 7. For data set B : sham = 10, GFP = 10, 5’ vector 2E+09 = 8, 5’ vector 2E+10 = 9, 3’ vector = 6, dual vector 2E+09 = 9, Dual vector 2E+10 = 10. For data set C, only mice for which 3 and 6 months data were available were included: sham = 7, GFP = 7, 5’ vector 2E+09 = 7, 5’ vector 2E+10 = 7, 3’ vector = 3, dual vector 2E+09 = 9, Dual vector 2E+10 = 7.
