# Supplementary material for: Assessment of AAV Dual Vector Safety in the Abca4−/− Mouse Model of Stargardt Disease
Source: Transl Vis Sci Technol. 2020 Jun 18;9(7):20. doi: 10.1167/tvst.9.7.20 (PMC7115835; doi:10.1167/tvst.9.7.20)
Supplement: Supplement 4 [file tvst-9-7-20_s004.pdf]

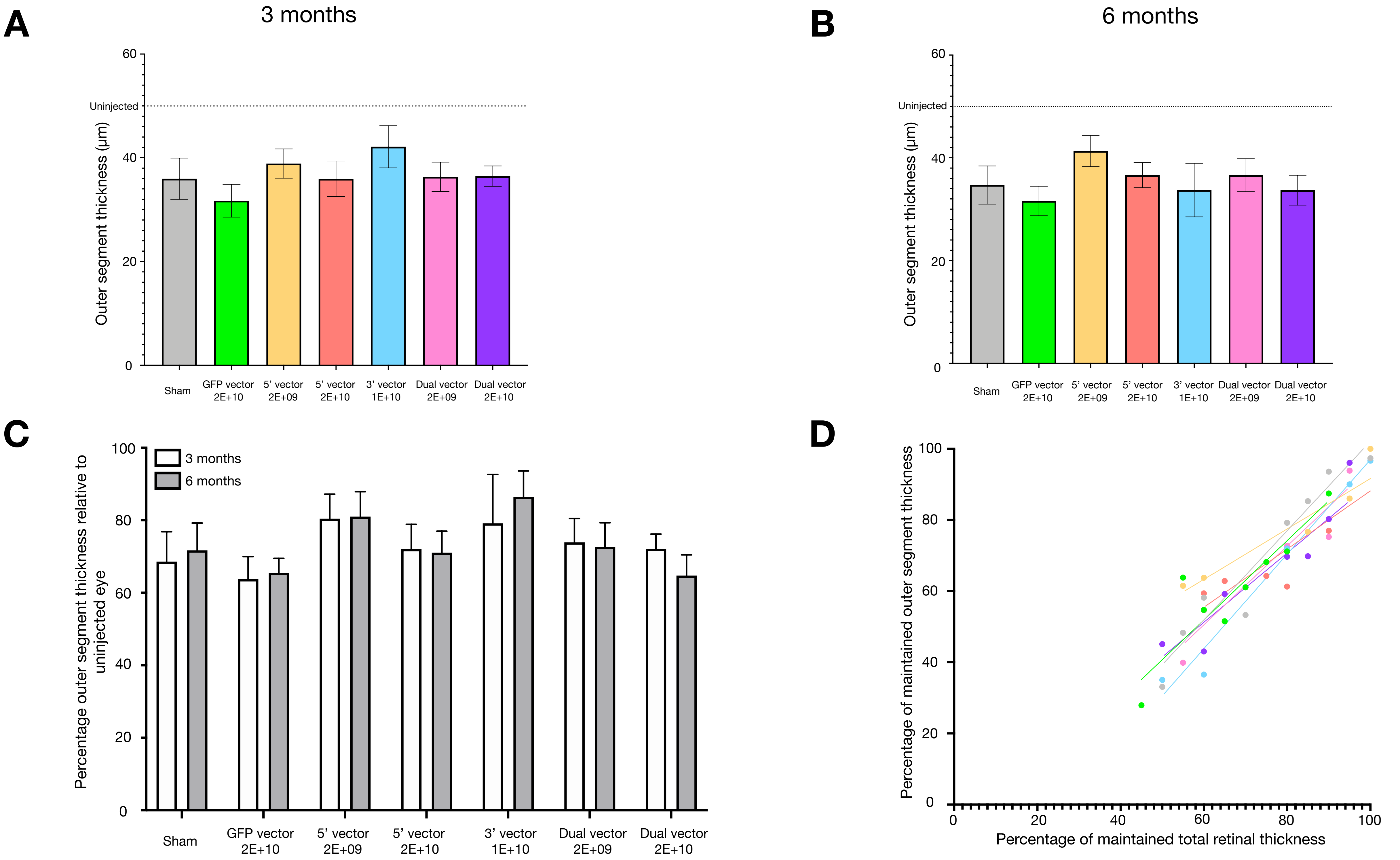

Supplementary Figure 4. Outer segment thickness measurements for the injected region of retina for all cohorts at 3 (A) and 6 months (B). The average outer segment thickness of the equivalent area of retina in uninjected eyes is marked for reference. Normalizing the measurements to paired uninjected eyes (C) revealed the changes in outer segment thickness were not significantly different between cohorts or time points (two-way ANOVA, injection material p=0.5832, time point p=0.7072, interaction p=0.1260) with no significant differences identified in multiple comparisons. Comparisons of the percentage of surviving total retinal thickness and outer segment thickness in the injected area of eyes revealed significant correlation at 6 months post-injection (D, see also Supplementary Table 3). For data set A : sham = 8, GFP = 7, 5' vector 2E+09 = 9, 5' vector 2E+10 = 7, 3' vector = 5, dual vector 2E+09 = 9, Dual vector 2E+10 = 7. For data set B: sham = 10, GFP = 10, 5' vector 2E+09 = 8, 5' vector 2E+10 = 9, 3' vector = 6, dual vector 2E+09 = 9, Dual vector 2E+10 = 10. For data set C, only mice for which 3 and 6 months data were available were included: sham = 7, GFP = 7, 5' vector 2E+09 = 7, 5' vector 2E+10 = 7, 3' vector = 3, dual vector 2E+09 = 9, Dual vector 2E+10 = 7.
