# Supplementary material for: Assessment of AAV Dual Vector Safety in the Abca4−/− Mouse Model of Stargardt Disease
Source: Transl Vis Sci Technol. 2020 Jun 18;9(7):20. doi: 10.1167/tvst.9.7.20 (PMC7115835; doi:10.1167/tvst.9.7.20)
Supplement: Supplement 5 [file tvst-9-7-20_s005.pdf]

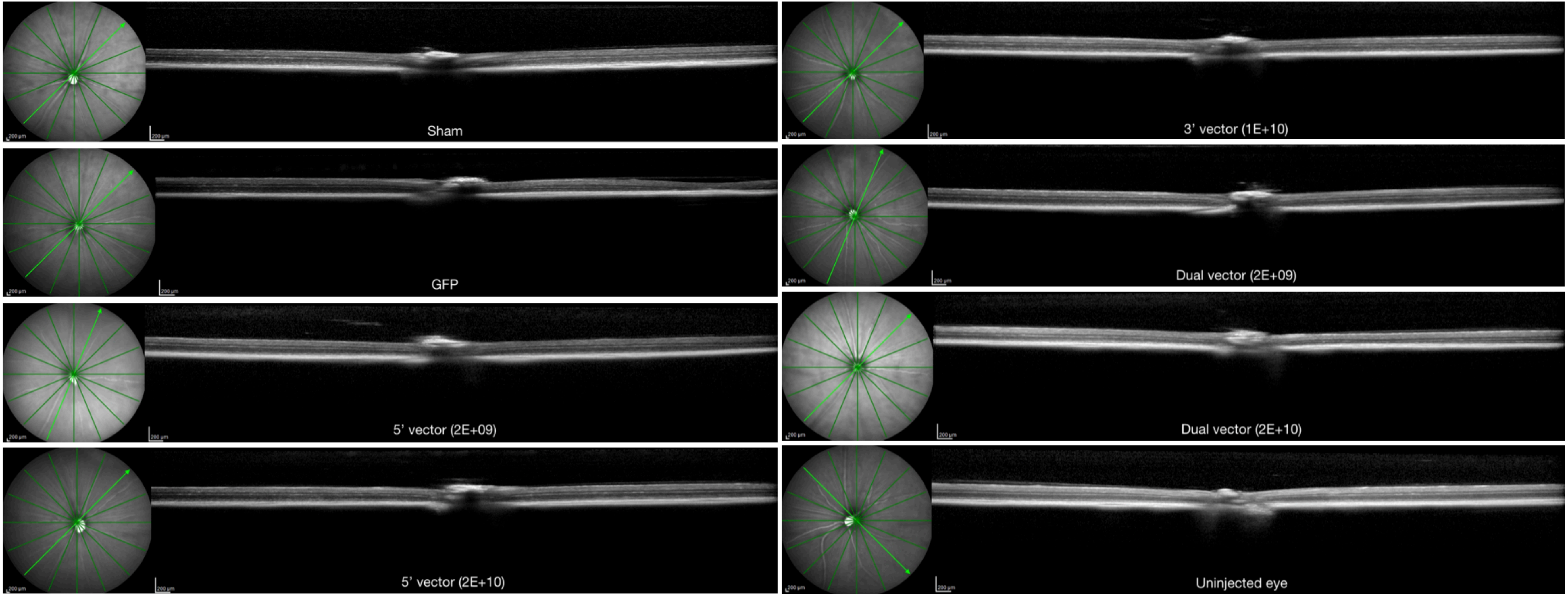

Supplementary Figure 5. Example OCT scans of injected eyes taken 6 months post-injection through the region of injection for each cohort.
