# Supplementary material for: Assessment of AAV Dual Vector Safety in the Abca4−/− Mouse Model of Stargardt Disease
Source: Transl Vis Sci Technol. 2020 Jun 18;9(7):20. doi: 10.1167/tvst.9.7.20 (PMC7115835; doi:10.1167/tvst.9.7.20)
Supplement: Supplement 6 [file tvst-9-7-20_s006.pdf]

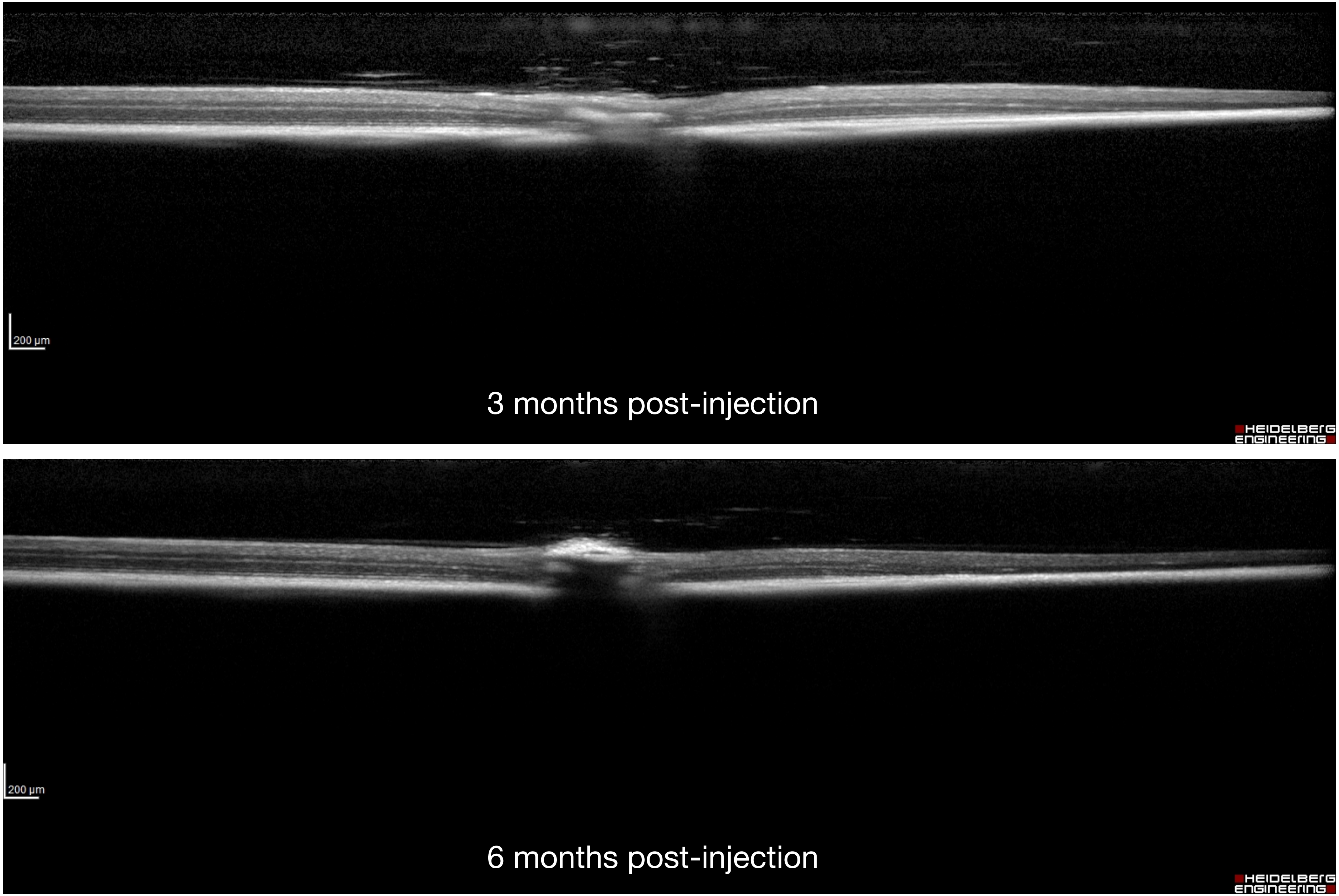

Supplementary Figure 6. Comparisons of retinal sections of an eye injected with 5’ vector 2E+10 showing cells in the vitreous at 3 months post-injection that were less apparent at 6 month post-injection.
