# Supplementary material for: Assessment of AAV Dual Vector Safety in the Abca4−/− Mouse Model of Stargardt Disease
Source: Transl Vis Sci Technol. 2020 Jun 18;9(7):20. doi: 10.1167/tvst.9.7.20 (PMC7115835; doi:10.1167/tvst.9.7.20)
Supplement: Supplement 7 [file tvst-9-7-20_s007.pdf]

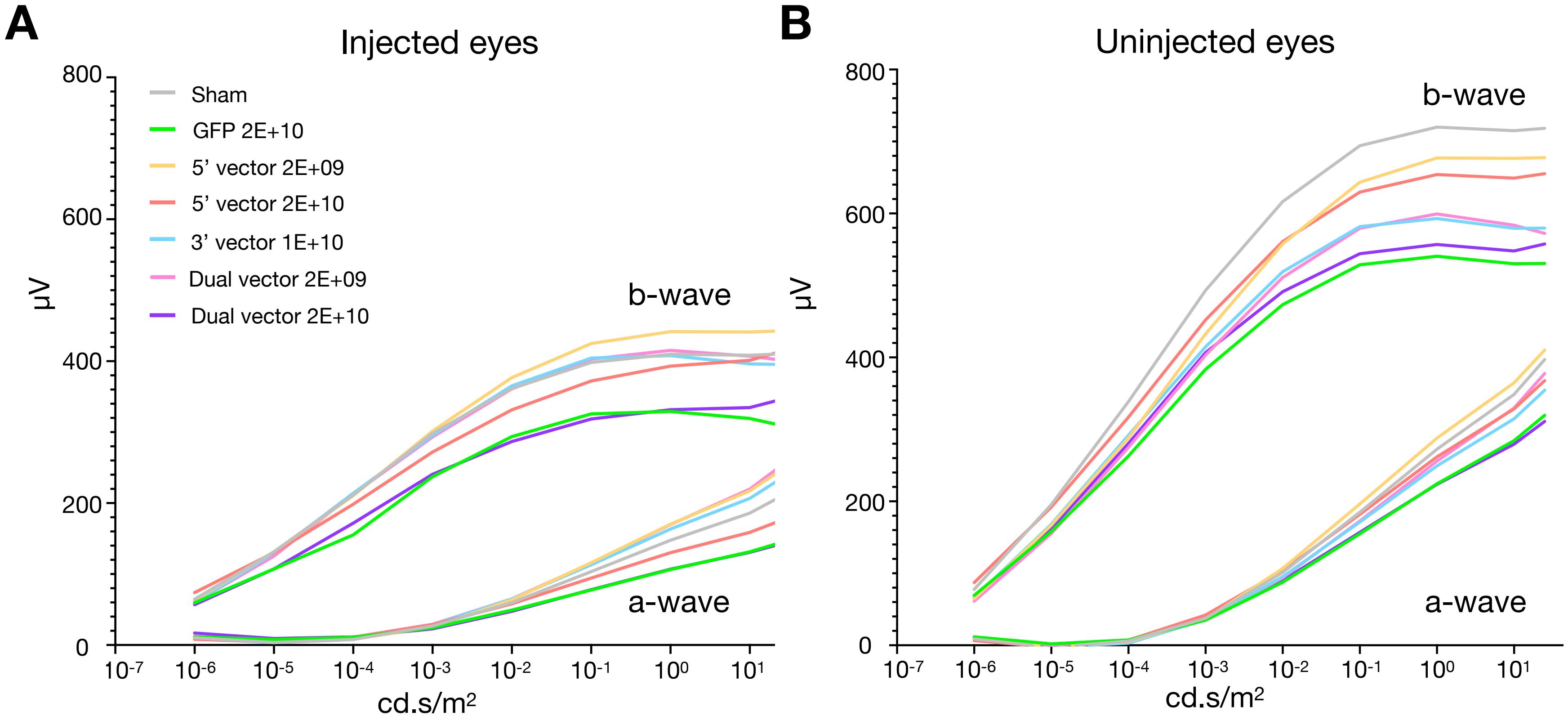

Supplementary Figure 7. Electroretinogram (ERG) response data 3 months post-injection. Smoothed average scotopic a- and b-wave response curves are shown for injected (A) and uninjected (B) eyes for all cohorts.
