# Supplementary material for: Assessment of AAV Dual Vector Safety in the Abca4−/− Mouse Model of Stargardt Disease
Source: Transl Vis Sci Technol. 2020 Jun 18;9(7):20. doi: 10.1167/tvst.9.7.20 (PMC7115835; doi:10.1167/tvst.9.7.20)
Supplement: Supplement 8 [file tvst-9-7-20_s008.pdf]

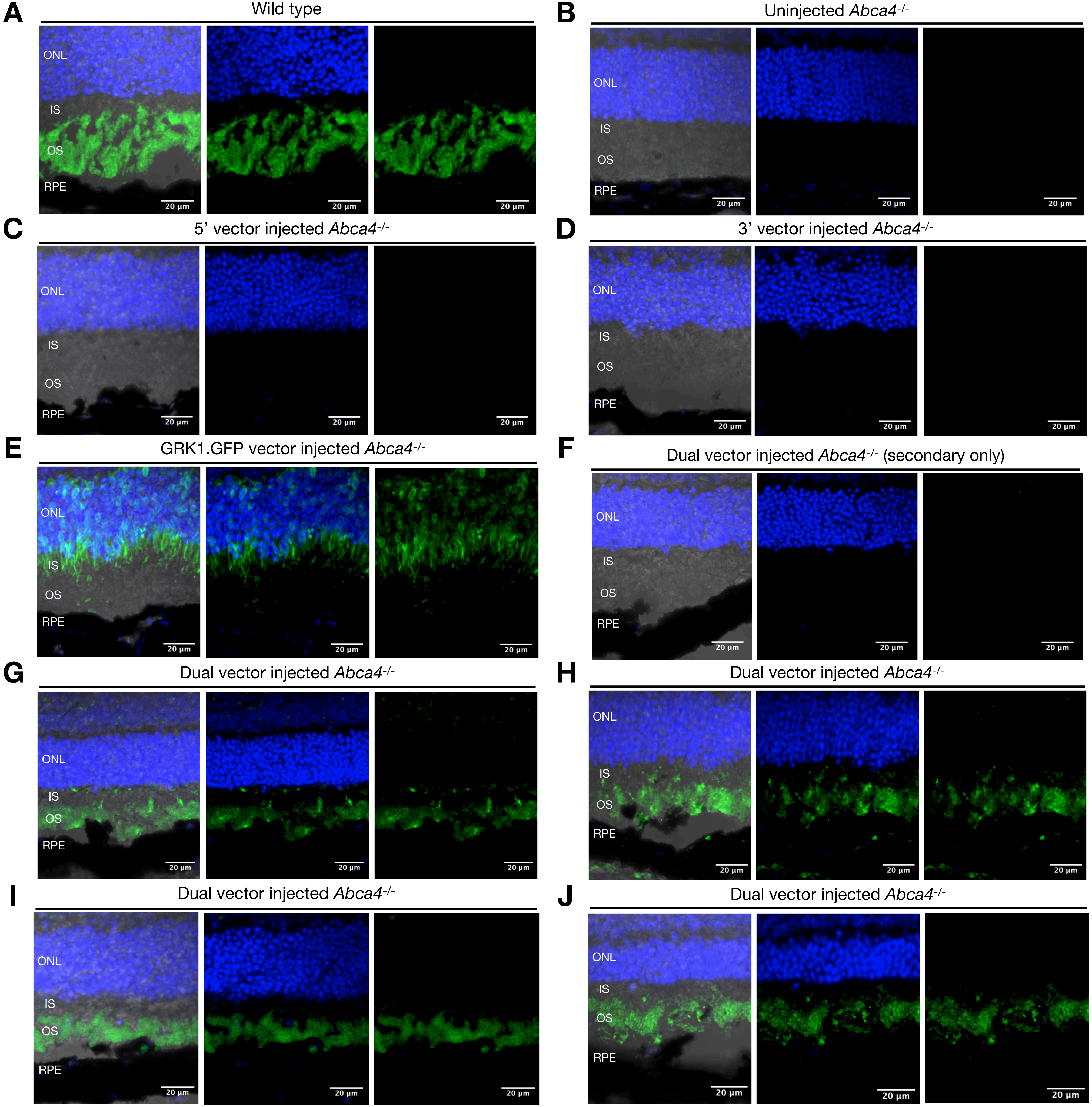

Supplementary Figure 8. ABCA4 staining (green) of eyes 6 months post-injection. (A) Detection of native Abca4 was achieved in the photoreceptor outer segments of wild-type mice with no detection in uninjected *Abca4*$^{-/-}$ eyes (B) or those injected with 5' vector (C), 3' vector (D) or the GRK1.GFP reporter vector (E). No staining was observed in dual vector injected eyes when using the secondary antibody only (F) but staining of ABCA4 was detected in the photoreceptor outer segments of dual vector injected eyes 6 months post-injection (G-J, images taken from three eyes).
