# Supplementary material for: Assessment of AAV Dual Vector Safety in the Abca4−/− Mouse Model of Stargardt Disease
Source: Transl Vis Sci Technol. 2020 Jun 18;9(7):20. doi: 10.1167/tvst.9.7.20 (PMC7115835; doi:10.1167/tvst.9.7.20)
Supplement: Supplement 9 [file tvst-9-7-20_s009.pdf]

Supplementary Table 1. Scotopic electroretinography protocol.

**Step**	**Trials**	**Inter sweep delay**	**Sweep length**	**Delay to next step**	**Description**	**cd.s/m²**
1	16	0	3,000	48,000	Single 0.5Hz	1E-06
2	16	0	3,000	48,000	Single 0.5Hz	1E-05
3	9	1,000	3,000	35,000	Single 0.5Hz	0.0001
4	9	1,000	3,000	35,000	Single 0.5Hz	0.001
5	9	5,000	3,000	67,000	Single 0.5Hz	0.01
6	4	13,000	3,000	51,000	Single 0.5Hz	0.1
7	4	29,000	3,000	99,000	Single 0.5Hz	1
8	4	61,000	3,000	195,000	Single 0.5Hz	10
9	1	0	3,000	3,000	Single 0.5Hz	25
10	25	0	500	12,500	Continuous 6Hz	0.01
11	25	0	500	12,500	Continuous 6Hz	0.1
12	25	0	500	12,500	Continuous 6Hz	1
13	25	0	500	12,500	Continuous 6Hz	3
14	25	0	500	12,500	Continuous 6Hz	10
15	25	0	500	12,500	Continuous 6Hz	10
16	25	0	500	12,500	Continuous 6Hz	10
