# Supplementary material for: Assessment of AAV Dual Vector Safety in the Abca4−/− Mouse Model of Stargardt Disease
Source: Transl Vis Sci Technol. 2020 Jun 18;9(7):20. doi: 10.1167/tvst.9.7.20 (PMC7115835; doi:10.1167/tvst.9.7.20)
Supplement: Supplement 10 [file tvst-9-7-20_s010.pdf]

Supplementary Table 2. Photopic electroretinography protocol.

**Step**	**Trials**	**Inter sweep delay**	**Sweep length**	**Delay to next step**	**Description**	**cd.s/m^2**
1	25	0	500	12,500	Single 0.5Hz	0.3
2	25	0	500	12,500	Single 0.5Hz	1
3	25	0	500	12,500	Single 0.5Hz	3
4	25	0	500	12,500	Single 0.5Hz	10
5	25	0	500	12,500	Single 0.5Hz	25
6	25	0	500	12,500	Continuous 20Hz	10
7	25	0	500	12,500	Continuous 30Hz	10
