# Supplementary material for: Assessment of AAV Dual Vector Safety in the Abca4−/− Mouse Model of Stargardt Disease
Source: Transl Vis Sci Technol. 2020 Jun 18;9(7):20. doi: 10.1167/tvst.9.7.20 (PMC7115835; doi:10.1167/tvst.9.7.20)
Supplement: Supplement 11 [file tvst-9-7-20_s011.pdf]

Supplementary Table 3. Correlation data for percentage of maintained outer segment thickness and percentage of maintained total retinal thickness in the area of injection at 6 months (data plot shown in Supplementary Figure 4D).

	**Sham**	**GFP 2E+10**	**5' vector 2E+09**	**5' vector 2E+10**	**3' vector 1E+10**	**Dual 2E+09**	**Dual 2E+10**
r	0.9665	0.8948	0.9549	0.8720	0.9887	0.9606	0.9459
P	<0.0001	0.0027	0.0114	0.0235	0.0014	0.0093	0.0013
